# Measuring bacterial oxygen consumption rate to probe metabolic signature and antimicrobial susceptibility

**DOI:** 10.1007/s00249-026-01834-7

**Published:** 2026-03-28

**Authors:** Chiara Scribani Rossi, Simone Angeli, Bruno Casciaro, Maria Rosa Loffredo, Maria Luisa Mangoni, Sharon Spizzichino, Giovanna Boumis, Manuel Espinosa-Urgel, Marzia Arese, Alessio Paone, Francesca Cutruzzolà, Serena Rinaldo

**Affiliations:** 1 Department of Biochemical Sciences “A. Rossi Fanelli”, Sapienza University of Rome, Rome, Italy; 2 Department of Biotechnology and Environmental Protection, Estación Experimental del Zaidin, CSIC, Granada, Spain

**Keywords:** Bacterial respiration, Antimicrobials, Oxygen consumption, Seahorse instrument

## Abstract

Quantitative metabolic profiling of bacterial communities provides biophysical insight into cellular respiration and energy transduction under controlled environmental conditions. The oxygen consumption rate (OCR) represents a direct, integrative readout of respiratory activity and has emerged as a sensitive phenotypic parameter for characterizing microbial physiology across different metabolic states. Here, we present an experimental protocol for measuring bacterial respiration using the Seahorse XFe96 Analyzer (Agilent), a microplate-based platform that enables high temporal resolution measurements of oxygen flux. Although originally developed for eukaryotic cell cultures, we adapted this technology to bacterial systems, allowing the simultaneous analysis of multiple strains in a medium-throughput format. The 96-well configuration, combined with sequential injection of up to four compounds, including metabolic substrates, stressors, and alternative electron acceptors, enables real-time perturbation of respiration and monitoring of dynamic OCR responses. To investigate bacterial respiration under physiologically and environmentally relevant oxygen tensions, OCR measurements were additionally performed under hypoxic conditions (5% O₂) by integrating the Seahorse XFe96 Analyzer with an oxygen-controlled glove box. This approach permits precise control of oxygen availability and extends OCR analysis to low-oxygen regimes relevant to chronic infections and aquatic or engineered environments. Overall, our results establish a robust and versatile biophysical platform for probing bacterial respiratory dynamics and for phenotypic screening applications.

**Supplementary Information:**

The online version contains supplementary material available at 10.1007/s00249-026-01834-7.

## Introduction

Bacterial respiration integrates cellular electron transport processes into a collective metabolic response that depends on oxygen availability and environmental constraints. Measuring the rate of bacterial respiration thus provides valuable insights into the metabolic status and adaptation of a bacterial community in response to environmental changes, both in the planktonic or in the biofilm states (Brutinel and Gralnick 2012; Jo et al. 2022; Scribani-Rossi et al. 2023). From a biophysical perspective, the oxygen consumption rate (OCR) represents a quantitative measure of respiratory fluxes emerging from the coupling between electron transport, redox balance, and energy conservation at the cellular level. OCR integrates the collective activity of respiratory chains within a population, providing a system-level readout that is sensitive to metabolic state, environmental constraints, and external perturbations. Importantly, respiration can be probed under controlled conditions, where oxygen availability acts as a tunable physical parameter shaping electron flow and energy dissipation. Time-resolved OCR measurements further allow the characterization of transient metabolic responses and steady-state regimes following defined perturbations. 

Profiling bacterial respiration is a central requirement across a broad range of basic and applied research fields. In synthetic biology, metabolic engineering of bacterial respiratory pathways is used to modulate key bioprocess parameters, including cell yield and productivity, with the aim of developing robust strains for biotechnological production (Kalnenieks et al. 2019; Wu et al. 2016). Bacterial respiration measurements provide a cultivation-independent approach to monitor bacterial accumulation in industrial pipeline systems (Toman et al. 2020). Biocorrosion and aerosol-mediated contamination, including *Legionella,* are among the major concerns in humid environments and real-time monitoring of oxygen consumption can help in identifying the appropriate disinfection measures (Paniagua et al. 2020; Toman et al. 2020). This approach has been successfully applied in the food industry to prevent microbial spoilage and foodborne disease, with the idea to develop a multi-parametric toxicity testing platform (Elisseeva et al. 2020). Beyond industry, measuring OCR in bacterial communities is crucial to understand the regulation of carbon fluxes in aquatic environments, as well as in wastewater treatment, where models of biological degradation processes must be carefully analyzed to design robust treatment systems (Guo et al. 2022; Pollard 2006). Finally, OCR determination represents a key parameter in biomedicine, where the growing burden of drug-resistant infections underscores the need for rapid and reliable methods to screen antimicrobial susceptibility in biological samples. To date, many efforts have been done to develop antibiotic susceptibility tests (AST) based on metabolic profiling rather than on cultivation (Heithoff et al. 2023; Jusková et al. 2021; Liu et al. 2021; Lopatkin et al. 1979; Lobritz et al. 2015; Yang et al. 2017).

A key advantage of OCR quantification is that it provides a growth-independent approach to bacterial population analysis, overcoming major limitations of culture-based methods, particularly for viable but non-culturable species (as in ex vivo samples). Growth impairment may arise from irreversible DNA damage, specific environmental requirements, dependence on symbiotic partners, or extremely slow growth rates. Moreover, facultative anaerobic bacteria often exhibit unchanged growth under reduced oxygen conditions due to a metabolic shift toward fermentation, while their respiratory activity is markedly altered. The limitation of growth-based methods has been only partly overcome by direct optical detection methods, such as flow cytometry (Nebe-Von-Caron et al. 2000). Although useful for community profiling, this single cell-based method is less useful for drug screening, where a community should be analyzed in full.

OCR therefore constitutes a direct and physically grounded observable to investigate bacterial metabolic dynamics beyond growth-based descriptors. Accordingly, multiple OCR-based methodologies have been developed to enable bacterial profiling and screening. Classical approaches, such as Clark’s electrodes, are limited by low sensitivity, signal drift, and probe fragility, while optical sensor–based techniques using fluorescence or luminescence have enabled OCR quantification with improved robustness, but only a few include the possibility to use a multi-well plate or a semi-automated platform (Almatrood et al. 2022; Bulock et al. 2021; Elisseeva et al. 2020; Jasionek et al. 2013; Jusková et al. 2021, 2024; Liu et al. 2021; Tahirbegi et al. 2017; Toman et al. 2020; Warkentin et al. 2007).

By contrast, OCR is routinely quantified in eukaryotic cell biology using the Seahorse XF Analyzers (Agilent), a microplate-based platform that provides high-resolution measurements of cellular respiratory activity and metabolic state in adherent cell cultures. Liu et al. have indeed acknowledged (Liu et al. 2021) that a Seahorse-based method designed to measure bacterial OCR could significantly contribute to the field. The platform enables medium-throughput measurements while allowing sequential, automated perturbation of the system through the injection of multiple compounds, thereby providing access to the dynamic response of oxygen consumption under controlled experimental conditions. A pilot experiment has been published in 2015 to investigate the antibiotic susceptibility in *E. coli* and *Staphylococcus aureus *(Lobritz et al. 2015), followed by more recent studies on *Pseudomonas putida* (Scribani-Rossi et al. 2023; Scribani-Rossi et al. 2024) and *Helicobacter pylori* (Maity et al. 2024), focusing on nutrients and antimicrobials, respectively. The last two systems have been successfully profiled by using Seahorse within the EU H2020 Molecular Scale Biophysics Research Infrastructures (MOSBRI) network at the Sapienza University of Rome (Italy) (HYP-ACB platform).

Here, we describe a detailed characterization of the methodology testing different bacteria, under different conditions of nutrients, oxygen, electron acceptors and stressors, to evaluate the robustness, the weaknesses, and the potential of this approach. By systematically assessing the flexibility and applicability of the approach under different experimental conditions we aim to providing the scientific community with a standardized and reproducible methodological framework.

## Experimental procedures

### Bacterial strains and growth media

*Escherichia coli* BW25113 was grown in M9 minimal medium supplemented with 1 mM MgSO_4_ (hereinafter M9*) plus 10 mM glucose (the last two solutions have been sterilized separately by filtration prior to addition to the autoclaved medium). *Pseudomonas aeruginosa* PA14 was grown according to (Chen et al. 2003) Glucose, 10 g/L; NH_4_Cl 6 g/L; KH_2_PO_4_, 0.7 g/L; NaCl, 0.5 g/L MgSO_4_ ·7H_2_O, 0.18 g/L; CaCl_2_, 0.01 g/L; MnCl_2_ · 4H_2_O, 0.01 g/L; FeSO_4_, 0.01 g/L; pH 5.5. The medium has been sterilized by filtration.

### Bacterial growth

Each growth per strain started from a single colony inoculated in the corresponding growth medium. *E. coli* was inoculated into 2 ml of SOC media, 2 h at 37 °C and 180 rpm; the preculture was diluted 1:100 in 10 ml of M9* plus glucose. The overnight culture was diluted to a final OD_600_ = 0.1 into 10 mL of fresh media and grown at 37 °C and 180 rpm until an OD_600_ ~ 0.25 was reached. *P. aeruginosa* was inoculated into 2 mL of growth media at 37 °C, after 24 h the culture was diluted 1:5 in fresh media (aerobic condition or “not induced”, NI) or fresh media plus 10 g/L of NaNO_3_ (denitrifying aerobic condition or “induced”, I) and let to grow overnight at 37 °C until reaching an OD_600_ = 0.1. An overview of the growth protocol for all the strains used in this work is reported in Figure S1A.

For *P. putida* assays on biofilm, a colony of KT2440 and KT2440 ΔlapAΔlapF (Martínez-Gil et al. 2010; Ruiz et al. 2021) were inoculated into 2 ml of LB and incubated overnight at 180 rpm and 30 °C. The day before, the strains were diluted in M9 minimal medium to an OD_600_ = 1 and subsequently diluted in LB medium to achieve final OD_600_ = of 0.045, 0.0225, and 0.0125. Biofilm growth and Seahorse plate seeding was, in this case, the same experimental phase and therefore it is described in this paragraph (see the next one for the other seeding procedures). A 15 µl volume from each dilution was seeded into a Seahorse plate, which was then incubated at 30 °C without shaking. The medium was then removed, the wells were washed twice with M9 medium and refilled with 200 µl of M9 medium containing 10 mM glucose.

### Plate coating and cell seeding

Before cells seeding, the Seahorse 96-well plate was coated by adding in each well poly-L-lysine (PLL) or collagen, as described in the manuscript. The PLL coating was performed by adding 15 μL of a 0.15 mg/mL solution of PLL hydrobromide (P6282, Sigma Aldrich) in water. The liquid was removed after 1 h and the wells were rinsed twice with 30 μL of ultra-pure water and plates were dried for 1 h under laminar flow. The collagen coating was performed by using 20 μL of a 1 × collagen solution in each well, previously prepared from a 20 × stock type I solution (Sigma C3867-1VL). The collagen solution was removed after 1 h, and the wells were dried for 1 h under laminar flow. Bacterial cultures were diluted at OD_600_ of 0.03, 0.02 or 0.01 with sterile water and 90 μL was used to seed the precoated plate. If reported, seeding and measurements were done under hypoxic conditions, using a Don Whitley (i2 workstation) hypoxic chamber (95% N_2_, 5% O_2_, 20% humidity) as indicated. For hypoxic experiments, the last column of the plate contained the sole medium to allow the addition of sodium sulfite (VWR 0628-500 g) during the run. Sodium sulfite (100 mM final concentration), loaded into the Seahorse cartridge, was used as a chemical oxygen scavenger to provide a ‘zero’ oxygen reference during the measurement. After seeding, plates were then centrifuged for 10′ at 4000 rpm and 90 μL of 2 × growth media was added to reach a total volume of 180 μL 1 × medium.

### Seahorse experiments

**Fig. 1:**
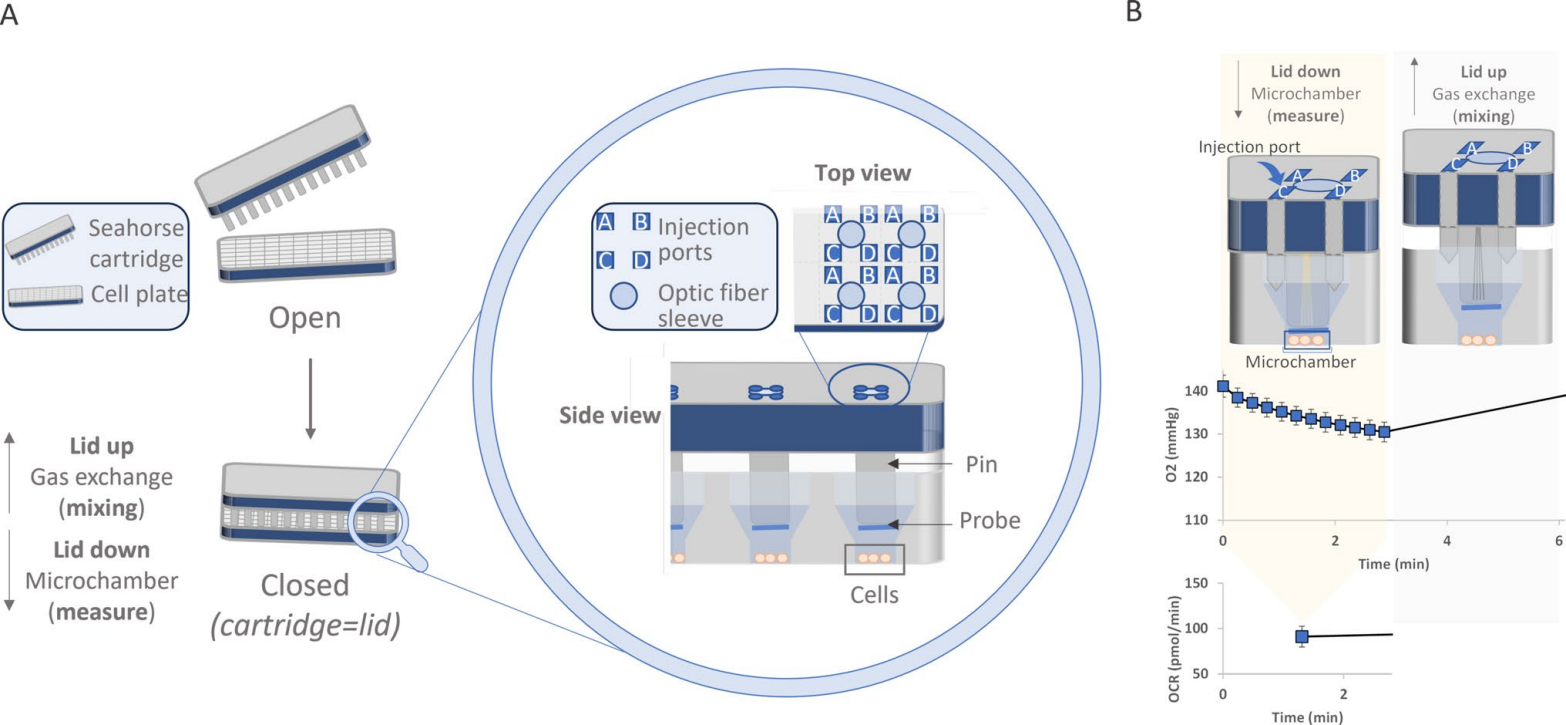
**A**) On the left, the Seahorse cartridge and cell plate in the open or in the closed state are shown (upper and lower panel, respectively). In the closed position, the cartridge is used as cell plate lid. The blow up includes a side view of the pin, coated with the oxygen probes, fitting the cell well. In this view, the longitudinal section of a micro-well of the cell plate plus the lid is reported; cells (orange ovals) are seeded as an attached monolayer and immerged into the medium (sky-blue in the Figure). Each pin fits with the narrow part of the cell micro-well. The corresponding top view is also reported, including a boxed legend; the top view shows the central hole serving optic fiber sleeve and 4 small injection ports for addition of chemicals. **B**) A cartoon of one well of Seahorse cell plate + cartridge is shown, including both the “Lid down” and “Lid up” states, corresponding to the measurement and mixing phase, respectively. In the “Lid down” state, the microchamber formed above each well allows the instrument to detect variations in oxygen levels (middle panel), which are used by the software to calculate the oxygen consumption rate (OCR, lower panel). Oxygen level fluctuations are detected by an oxygen-sensitive fluorophore positioned on the cartridge sensor pin and exposed to the surrounding medium

Bacterial respiration was measured using the Agilent Seahorse XFe Analyzer (hereinafter Seahorse) instrument in the MOSBRI EU Infrastructure – HypACB facility at Sapienza University of Rome (Italy). Briefly, cells are seeded on a dedicated 96-wells plate which can be closed with a special disposable lid, named cartridge, bearing 96 pins (one per well); each pin tip is covered with a layer of oxygen (and pH, not applicable for this analysis) fluorescence probe(s). The optic fibers of the instrument fit in each pin, to detect the fluorescent signal/variation occurring during measurement. The oxygen probe detects the oxygen levels over time, which are used to calculate the OCR (Fig. 1).

Each well has a bottleneck shape (longitudinally, Fig. 1, lower panel, side view) and the pin fits in the narrow section of the well, once the plate is covered by the lid; the instrument can automatically move the lid up and down to alternate open and closed state of the plate. In the closed state (Lid down in the Fig. 1), the almost gas-tight fit of the pin creates a microchamber where cell respiration (i.e. oxygen consumption) can be measured, since no gas exchange with the medium can occur; the OCR is then automatically calculated from these data.

After a user-defined interval, the instrument moves up the lid (Lid up in the Fig. 1A), the medium is mixed, allowing oxygen and nutrients equilibration with the rest of the medium occupying the large section of the well (200 µl in total). In this phase no measurements occur, but the baseline oxygen levels should be restored (more details are reported in Fig. 1B).

According to the manufacturer’s instructions, the Seahorse sensor cartridge was hydrated by adding 200 µL of Seahorse calibrant solution and incubated overnight at the same temperature used for the assay and cell growth. Before running the experiment, additives were loaded (20 μL of 10 × stock solution in 1 × medium) into the injection port A of the Seahorse cartridge; nutrients’ stock solutions were: 10 mM glucose, 10 mM arginine, 10 mM pyruvate and 10 g/L NaNO_3_. Peptides were dissolved in water and the final concentration was 0.05 µM, 0.5 µM, 5 µM and 50 µM.

Experimental set up was set with 3 min of mixing (lid up) followed by 3 min of measurements (lid down). After 3 baseline measurements, drugs or nutrients were automatically injected into each well. Measurement of bacterial respiration under hypoxic conditions was carried out into the Don Whitley (i2 workstation) hypoxic chamber (95% N_2_, 5% O_2_, 20% humidity). Given that bacterial growth may occur during prolonged measurements (although detachment has not been observed even at prolonged time of acquisition), we propose to use OCR for quantitative evaluation up to 45’ upon carbon source supplementation.

Experimental data have been calculated using the dedicated Wave software, provided by the vendor. A specific release for hypoxic measurements has been used for the experiment run under hypoxic conditions. According to the vendor instructions, XF Hypoxia Assay requires the use of sodium sulfite (a chemical oxygen scavenger) to provide a “zero” oxygen reference. This reactant is injected during the assay into a dedicated lane of wells containing XF Calibrant. This lane is therefore used to check the set oxygen tension (i.e. 5%) and the zero,

### Fluorescence detection of P. putida biofilm formation

A colony of *P. putida* strains KT2440-GFP (Martínez-Gil et al. 2010; Ruiz et al. 2021) and KT2440 ΔlapAΔlapF-GFP (Koch et al. 2001) was inoculated into 2 ml of Luria–Bertani (LB) medium and incubated overnight with shaking at 180 rpm at 30 °C. Strain KT2440-gfp was grown in the presence of Kanamycin (30 µg/ml), while strain KT2440 ΔlapAΔlapF-gfp was cultured with both Kanamycin (30 µg/ml) and Gentamycin (50 µg/ml). The day before, the strains were diluted in M9 minimal medium to an OD of 1 and subsequently diluted again in LB medium to achieve final OD of 0.09, 0.045, and 0.0225. From each dilution, 15 µl were seeded in a Seahorse plate and the plate was incubated at 30 °C without shaking. After incubation, the medium was removed, the wells were washed twice with M9 medium, and GFP fluorescence was detected using an Agilent BioTek Cytation 5 cell imaging multimode reader. The analysis was performed using a GFP light cube with an excitation filter of 469/35 nm and an emission filter of 525/39 nm. These strains were not used for Seahorse (due to the intrinsic fluorescence), but a Seahorse plate was used for seeding imaging, given the peculiar shape and volume of the well plate.

## Results and discussion

### Effect of coatings on OCR

We performed real-time bacterial respiration measurements using extracellular flux analyzer XFe96 Seahorse instrument (Agilent, hereinafter Seahorse).

Cells must be seeded in the narrow part of the well, attached at the bottom, to allow the instrument to alternate measurements (lid down, Fig. 1) and mixing (lid up, Fig. 1) phases without any cell detachment; cells resuspension during alternate phase leads to vary the number of cells monitored during the measurement phase (Lid down), thus hampering a comparable analysis among the replicates. We have tested two different coating strategies to promote bacterial adhesion: i) PLL, according to (Lobritz et al. 2015; Scribani-Rossi et al. 2023; Scribani-Rossi et al. 2024) and ii) collagen (Maity et al. 2024). In this work we present the coating optimization procedure used for *E. coli* BW25113, but a similar experimental design has been carried out for all the bacterial strains assayed in this work (summarized in Table 1).

**Table 1.** Coating and OD_600_ selected to seed bacterial cell in the Seahorse cell plate

Bacterial strain	Coating	OD_600_	Reference
*P. putida* KT2440 (normoxia)	Poly-Lys	0.02	Scribani-Rossi et al. 2023; Scribani-Rossi et al. 2024
*H. pylori* ATCC 700392 (hypoxia, 5% O_2_)	Collagen	0.6	Maity et al. 2024
*E. coli* BW25113	Poly-Lys	0.02	This work
*P. aeruginosa* PA14	Collagen	0.02	This work
*P. stutzeri* ZoBell MK21	Poly-Lys	0.01	This work

The yield and robustness of each adhesion medium were assessed using a Seahorse experiment, in which three different OD_600_ values were seeded on two different surface coatings. An effective coating should ensure a linear dependence of the OCR *vs* OD_600_ seeded; at the same time, an optimal cell dilution can be chosen based on the absolute OCR values, depending on the planned experimental design (see below). Values below 20 pmol/min are considered too low for reliable quantitative analysis/comparison. Prolonged measurements have been done to evaluate the robustness of adhesion during the mixing phase of the Seahorse measurements. If detachment occurs, highly scattered OCR are expected among technical replicates, due to variability of the number of cells retained in the microchamber (“lid down”, Fig. 1).

**Fig. 2:**
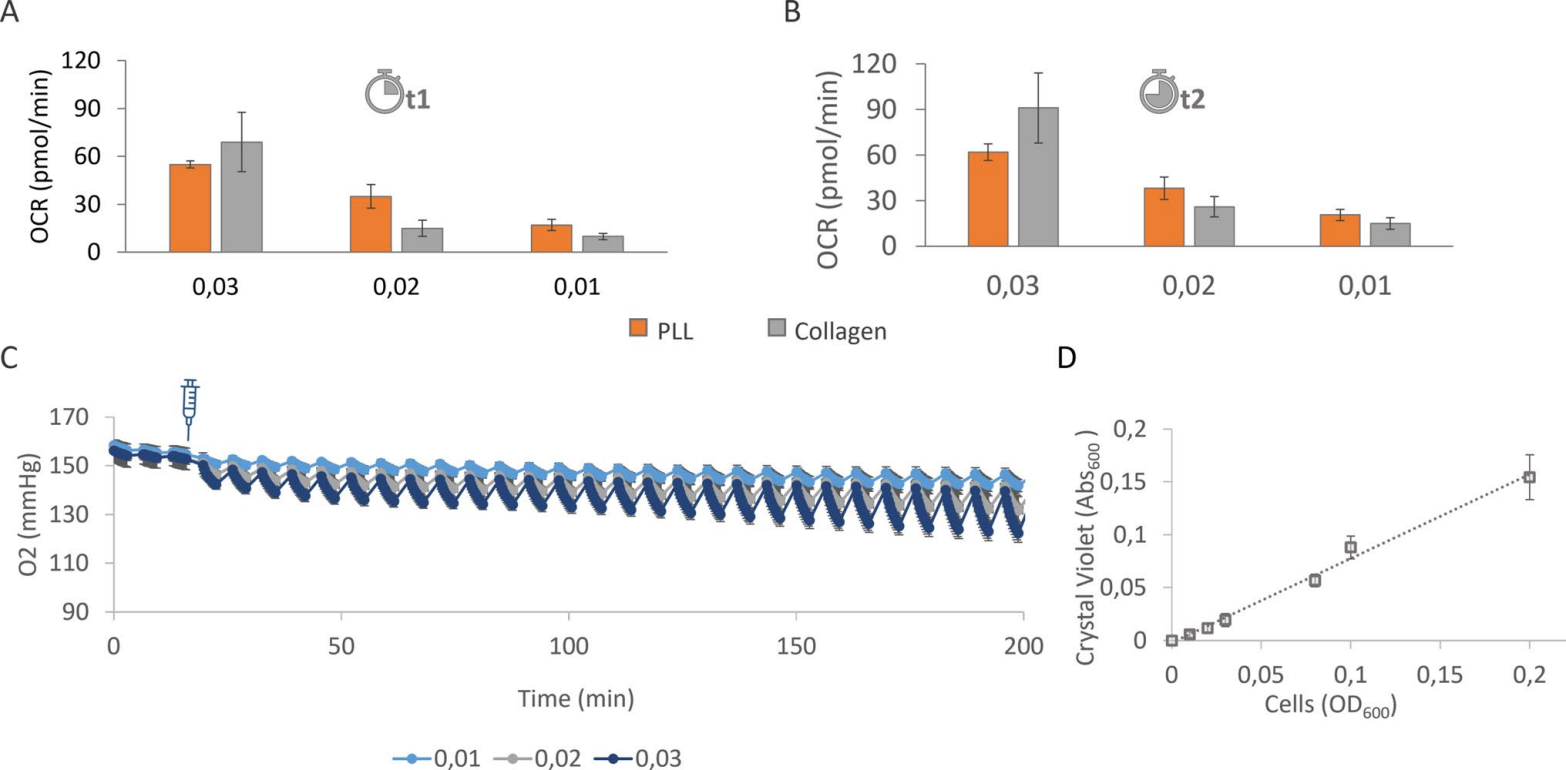
OCR values measured for E. *coli* BW25113. Coating and OD_600_ optimization were done by seeding three different OD_600_ on both PLL or Collagen coating (orange or grey in the Figure, respectively). OCR values refer to two different times after glucose supplementation (after 3’, t_1_ in panel A and 39’, t_2_ in panel B). X-axes values refer to the three OD_600_ assayed. Optimization experiment was done including at least 5 replicates. (**C**) Respiratory activity of 3 different dilution of seeded *E. coli* BW25113 (i.e. OD600 0.01, 0.02, 0.03 in the Figure); the oxygen level is reported, while the calculated OCR at a given time are reported in panel A. Oxygen levels were measured every 3 min alternated with 3 min of mixing for gas exchange (see Figure S1for technical details); the syringe indicates the point at which glucose was injected by the instrument. (**D**) Linear dependence of the absorbance of the Crystal violet assay as a function of the OD_600_ of seeded cells in the corresponding sample

Three different cell dilutions (reported as OD_600_) were seeded on the two different coating materials, i.e. PolyLysine (PLL) or Collagen; the corresponding OCR after 3’ of glucose supplementation (through injection port A) is reported in Fig. 2A. *E. coli* BW25113 basal OCR, with no carbon supplementation, is negligible regardless the seeding cell dilution and therefore not considered in this analysis. OCRs recorded 39’ after glucose supplementation are also shown (Fig. 2B) to evaluate the robustness of coating over time. According to our results, we selected for *E. coli* BW25113 the PLL coating and OD_600_ of 0.02 as the optimal, since the OCR falls in a good range (~ 20–30 pmol O_2_/min) and the oxygen consumption is not so avid to miss the baseline recovery over prolonged times (Fig. 2C). Crystal violet staining was carried out by seeding different dilutions of *E. coli* BW25113, to verify that the selected OD_600_ falls within a range where the in-solution OD_600_ *vs* attached cells has a linear dependence (Fig. 2D).

The OD_600_ range identified is optimal for seeding and measurement procedures; within this range, the exact value can be selected according to the experimental design and the aim of the analysis, including prolonged time analysis or the evaluation of the effect of additives/drugs.

### Effect of carbon sources on OCR

Very recently, Seahorse measurements were successfully used to profile the effect of different carbon sources on the OCR of *P. putida* and its selected genetic mutants (Scribani-Rossi et al. 2023; Scribani-Rossi et al. 2024). In that case, the study was focused on L-arginine, a multivalent metabolite serving both Carbon and Nitrogen source (Scribani Rossi et al. 2022), compared to glucose.

**Fig. 3:**
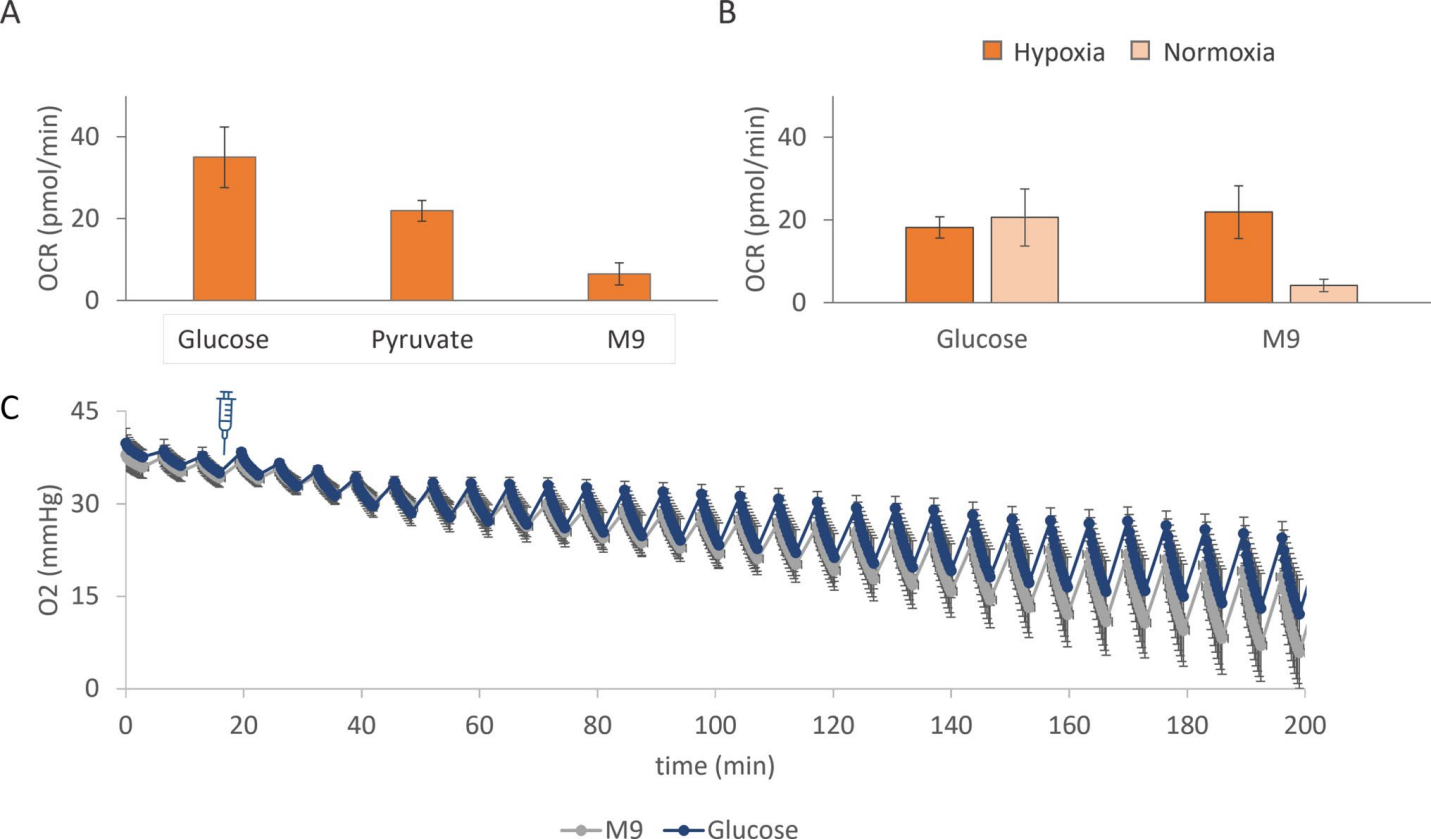
(**A**) The effect of carbon source on OCR was evaluated by comparing supplementation of glucose or pyruvate against the sole M9 minimal medium. In the graph the OCR value recorded upon addition of the corresponding carbon source, shown in the X-axes. M9 supplementation was used as control. (**B**) Glucose supplementation was profiled under both normoxic and hypoxic conditions at 5% O_2_ (light and dark orange, respectively) and compared to the OCR recorded in the presence of the sole M9. It is not excluded that the observed effect of hypoxia on OCR is a fast adaptative response due to the short exposure to low oxygen tension; it is likely that different protocols may lead to different effects on OCR. Experiments in panel (**A**) and (**B**) were done twice with at least 4 replicates for each sample; in the Figure a representative experiment is shown, including the average of 4 replicates ± SD. (**C**) The oxygen levels of the hypoxic respiratory activity reported in (**B**) are also showed. All these experiments were carried out with *E. coli* BW25113; oxygen levels were measured every 3 min alternated with 3 min of mixing for gas exchange (see Figure S1 for technical details); the syringe indicates the point at which Carbon source (or control) was injected by the instrument (i.e. Glucose or M9, respectively)

Here we compare the OCR measured in *E. coli* BW25113 in the presence of glucose and pyruvate. As reported in Fig. 3A, both glucose and pyruvate supplementation (10 mM each) stimulate the oxygen consumption as compared to the control sample (M9 medium without Carbon source, Fig. 3A), with the glucose yielding a slightly higher OCR than pyruvate. This pilot experiment confirms that both Carbon sources boost OCR in the same time window (upon supplementation); moreover, the metabolic potential of each Carbon source can be evaluated using Seahorse for quantitative analysis. Profiling pyruvate-dependent metabolism is useful for bacterial chemicals biosynthesis, being its metabolism (and that of its derivatives) attractive as an efficient and cost-effective alternative to traditional chemical synthesis method (Causey et al. 2004; Moxley and Eiteman 2021; Soma et al. 2022). Metabolic engineering trials to improve pyruvate accumulation may take advantage of a rapid screening of fuel metabolism to create industrially relevant and economically feasible bioprocesses.

### Effect of hypoxia on OCR

As mentioned above, bacterial bioenergetics has a huge impact on both biotechnological and biomedical fields, as a tool to improve the production yield and as a potential target for novel antimicrobials (Borisov et al. 2021; Cook et al. 2014).

Literature data indicates that bacterial bioenergetics often involves hypoxic conditions which are relevant not only in many infections and contaminations (Schaffer and Taylor 2015), but also in the bioremediation and industrial field. In industrial biotechnology, applications under oxygen-limited conditions have been explored to counteract the low productivity of aerobic processes (Pacheco et al. 2018). In clinical settings, the replacement of hypoxia with oxygen carriers has been proposed as anti-biofilm strategy (Hu et al. 2020), while the use of anaerobic bacteria as *chassis* to deliver antitumoral molecules has been tested as bacterial therapy to target the typical hypoxic environment of tumors (Chen et al. 2018; Samadi et al. 2021).

Given the relevance of hypoxia in shaping the bacterial metabolic signature, we have measured *E. coli* OCR at 5% O_2_, a condition reported in gut infections (Ding et al. 2001; Han et al. 2021). As shown in Fig. 3B, the OCR with or without glucose, as carbon source, is very similar despite the absence of carbon supplementation (M9 sample). The avidity of oxygen consumption is huge, and the sample fails to recover 5% O_2_ baseline (~ 40 mmHg) over time (Fig. 3C).

This phenotype can be due to an adaptive, stress-induced response to maintain ATP production efficiency when oxygen is scarce. Under these conditions, the expression of very efficient high-affinity terminal oxidases is induced; moreover, oxygen consumption could be also due to oxygen scavengers required to counteract the unbalancing between electron donors and acceptors (Borisov et al. 2021; Kawakami et al. 2010; Poole et al. 1996). Oxygen avidity was also observed in *P. putida*, known for its obligate aerobic nature (which discourages its exploitation as such in synthetic biology (Kampers et al. 2021)). *P. putida* oxygen consumption under hypoxia was so high that a 1:10 cell dilution was necessary to record a reliable OCR (although with a poor baseline recovery at prolonged time) (Figure S2).

Considering these results, we conclude that the Seahorse method can be used with bacteria under hypoxic conditions, although an ad hoc optimization of cell density may be required, which may be different from that found optimal for normoxia.

### Effect of electron acceptors on OCR

As mentioned above, the study of bacterial metabolism should consider the O_2_ levels found in the environment from which that community belongs to; nevertheless, a complete picture of the “redox zonation” should include the effect on metabolism of possible alternative electron acceptors overlapping with suboxic zone (Canfield and Thamdrump 2009). We therefore profiled OCR in the presence of an electron acceptor alternative to O_2_, under normoxic conditions, to evaluate whether the Seahorse system is sufficiently sensitive to detect these variations from a quantitative point-of-view.

**Fig. 4:**
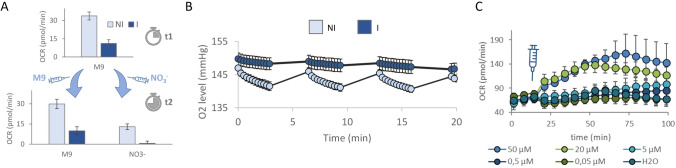
OCR of *P. aeruginosa* PA14 under different conditions. (**A**) Upper panel, the basal respiration (t_1_ = 18’) of *P. aeruginosa* PA14 grown in the presence of 0,1% NO_3_^−^, to induce aerobic denitrification (“I” sample in the Figure) is shown in comparison to the non-induced sample (“NI”). In the lower panel, the effect of NO_3_^−^ supplementation on both samples described in the upper panel is shown (t_2_ = 24’, the first point after supplementation) in comparison to M9 addition. (**B**) O_2_ levels of the experiment reported in panel (**A**). (**C**) The effect of the AMP Esc(1–21) at different concentrations (0.05–50 μM) on OCR was assayed upon supplementation during Seahorse experiment. Since the peptide was dissolved in water, water addition was used as control. Experiments were done twice with at least 5 replicates for each sample; in the Figure a representative experiment is shown, including the average of five replicates ± SD

The alternative respiratory condition selected was denitrification, i.e. the dissimilative nitrate reduction into gaseous dinitrogen, where nitrate acts as an electron acceptor. This pathway is inducible and is considered an anaerobic/microaerobic process, although more recently its contribution as an aerobic process has been acknowledged (Hao et al. 2022). We tested the effect of nitrate on *P. aeruginosa* PA14, a reference system for studying denitrification and biofilm (Jensen et al. 2017). Nitrate was used in the growth to induce the aerobic denitrification pathway (named induced, “I” in Fig. 4, in comparison with non-induced, “NI” sample). The induction of the denitrification was verified by western blot, showing basal expression and increased expression of nitrite reductase cd_1_NiR, in the “NI” and “I” samples, respectively (Farver et al. 2009) (Figure S3A). As shown in Fig. 4A, upper panel, the “I” sample, seeded with the nitrate-free medium, shows a lower basal OCR than the “NI” sample. This data indicates that the activation of the denitrification pathway competes with O_2_ for the electron flow, thus yielding a reduced aerobic respiration (Fig. 4B for O_2_ level). This effect is recapitulated upon NO_3_^−^ addition during the measurement, with “I” sample reaching very low OCR (Fig. 4A, lower panel). A similar trend was observed after seeding both “NI” or “I” samples with NO_3_^−^ ab initio, thus confirming that respiratory NO_3_^−^ reduction occurs very quickly (Figure S3B). On the other hand, NO_3_^−^ addition during the measurement has no effect on *P. stutzeri* MK21, a denitrifier whose denitrification is known to occur only under hypoxic or anaerobic conditions (Miyahara et al. 2010) (Figure S3C).

These findings indicates that this method can be used to highlight the versatility of electron acceptors used in different biological processes and conditions.

### OCR measurements to evaluate the response to antimicrobial peptides

OCR measurements can be used to assess antimicrobial susceptibility, and the 96-well format of the Seahorse platform enables the screening of multiple compounds and concentrations. Here, we measured the OCR of *P. aeruginosa* PA14 following exposure to antimicrobial peptides (AMPs) to evaluate the flexibility of the Seahorse-based approach. Membrane-active cationic AMPs are known to disrupt the integrity of the bacterial cytoplasmic membrane, which is expected to impact respiratory activity in a dose-dependent manner, although the temporal dynamics of the response remain difficult to predict (Luca et al. 2013; Mangoni et al. 2024). As a proof of concept, we quantified OCR variations upon treatment with the frog skin-derived AMP Esc(1–21), which exhibits well-documented anti-*Pseudomonas* activity (Casciaro et al. 2020; Loffredo et al. 2017). As reported in Fig. 4C, the addition of Esc(1–21) leads to dose-dependent changes in OCR, with concentrations of 20 and 50 μM significantly affecting oxygen consumption over time. In comparison, the effect of sub-micromolar concentrations was negligible, in line with the published minimal inhibitory concentration (MIC) (Luca et al. 2013). The larger OCR error bars at later time points in the 50 μM sample likely reflect increased cell detachment, potentially associated with cellular injury.

As previously mentioned, the development of an OCR-based platform for screening antimicrobials is desired, as a valid alternative to the growth-based approach (Jusková et al. 2021; Liu et al. 2021). This pilot experiment demonstrates the potential of the Seahorse platform as a time-efficient method for OCR-based antimicrobial screening and for probing the mechanistic effects of membrane-active antimicrobials. Moreover, compound libraries and antimicrobial susceptibility testing of clinical isolates, benchmarked against reference strains, may be implemented in a medium-throughput format.

### OCR measurements on biofilm

To evaluate the feasibility of using Seahorse to measure the OCR on biofilm, we have set a pilot experiment with ad hoc* P. putida* strains. We addressed two main concerns: (i) whether *P. putida* biofilm could form and grow on the bottom surface of the specialized wells used in the Seahorse plate; and (ii) whether the biofilm could withstand the mixing cycles associated with each Seahorse measurement without detaching over time.

**Fig. 5:**
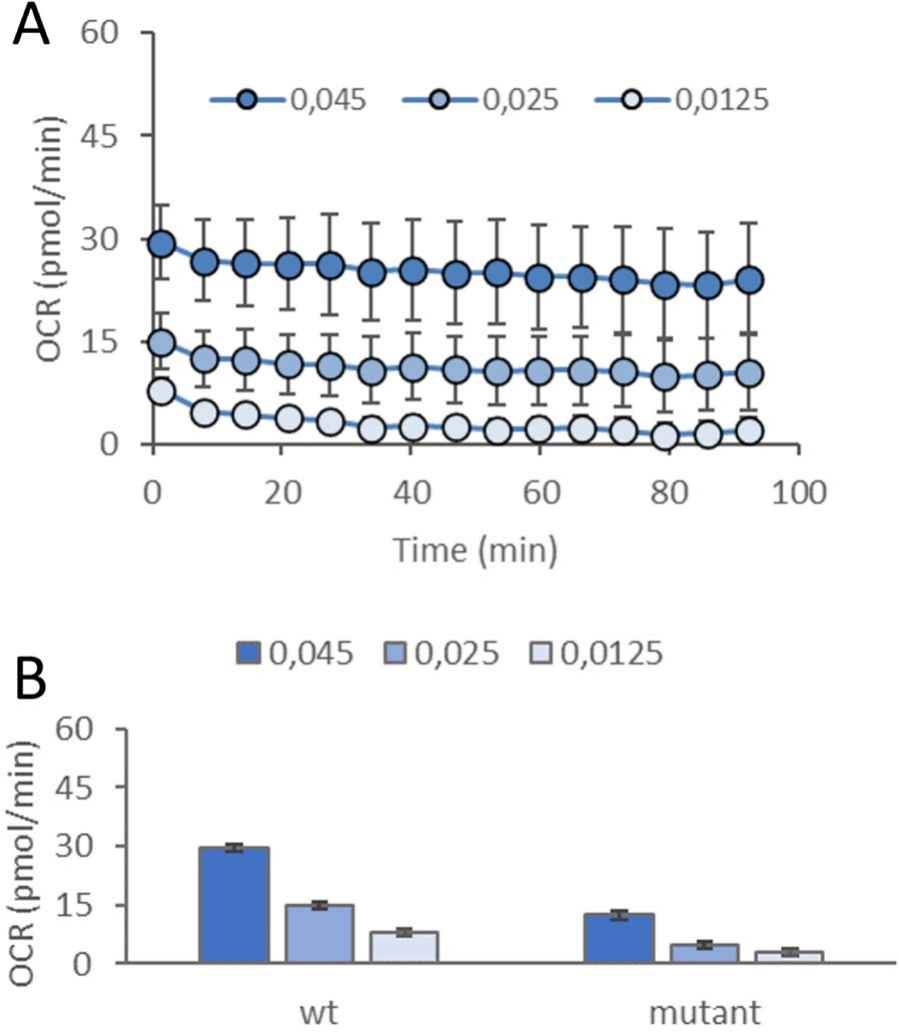
OCR of *P. putida* biofilm. (**A**) Three different OD_600_ (namely 0.045, 0.0225, 0.0125) of KT2440 strain were seeded and biofilm was allowed to grow. OCR was then measured over time. Lack of scattering at prolonged time indicates that no detachment has occurred. (**B**) Basal OCR of experiment reported in (**A**) was compared to the basal OCR of KT2440 ΔlapAΔlapF mutant seeded under the same conditions (wt and mutant in the Figure, respectively). Despite reduced biofilm formation, the signal measured in the mutant remains dependent on the initial seeding cell density

For the first point, it was crucial to avoid overaccumulation of biofilm, since the microchamber created during measurement needs room for the liquid phase. To probe this, we used a strain expressing the GFP protein (KT2440-GFP), seeded without any coating on a Seahorse plate; three different OD_600_ dilution were seeded, to appreciate a dependence of fluorescence signal in response to different cell dilution. This signal was evaluated by optical measurements, as shown in Figure S4A. The fluorescence signal is indeed dependent on OD_600_ dilution; the same seeding procedure carried out with the KT2440 ΔlapAΔlapF-GFP mutant strain (showing dramatically reduced biofilm formation) leads to a dramatic reduction of fluorescence signal (Figure S4B). This data was used to select the OD_600_ dilution range to run the Seahorse assay. In this case the same strains (i.e. KT2440 and KT2440 ΔlapAΔlapF), but lacking the GFP expression, were analyzed. As depicted in Fig. 5, following the Seahorse experiment, the OCR was i) dependent on seeded OD_600_ (Fig. 5A); ii) dependent on the capability of the strain to form biofilm (Fig. 5B); iii) stable over prolonged time, indicating that the biofilm-mediated surface attachment is sufficiently strong to sustain the mixing phase, without the need for coating (as required for the planktonic counterpart, Fig. 5A). Although preliminary, this data encourages future optimization for biofilm metabolic profiling.

## Conclusions

Biophysical research has traditionally relied on purified proteins to dissect molecular mechanisms. However, an increasing number of biologically relevant processes, particularly those involving respiration, redox homeostasis, and membrane-associated pathways, depend on complex interactions that are difficult to preserve outside the native cellular context. In such cases, probing protein function at the level of the whole microorganism becomes essential, especially under physiologically relevant conditions such as low oxygen availability or biofilm growth.

In this study, we systematically evaluated the applicability of Seahorse-based OCR measurements to bacterial systems across multiple experimental conditions. Although originally developed for profiling mitochondrial respiration in eukaryotic cells, we demonstrate that this approach can be effectively adapted to bacteria when appropriate surface coatings are employed. The sensitivity of the method is sufficient to resolve respiratory changes induced either prior to the assay (e.g., nitrate supplementation) or dynamically during measurement, enabling real-time monitoring of metabolic responses.

A key strength of this platform lies in the combination of medium-throughput capacity and dynamic perturbation within a 96-well format, allowing parallel screening of nutrients, stressors, and antimicrobial compounds across multiple concentrations. While bacterial growth may occur during acquisition, the comparative nature of OCR profiling ensures that deviations from reference respiratory traces remain highly informative of metabolic perturbations. Given the central role of respiration in biofilm organization and persistence (Hall and Mah 2017) as well as in the maintenance of tolerant phenotypes in chronic and antibiotic-resistant infections (Orman and Brynildsen 2015), extending OCR measurements to bacterial biofilm communities represents a compelling future direction.

Overall, the methodological framework presented here provides biophysicists with a robust and flexible experimental approach to investigate bacterial protein function in the cell, thereby bridging classical biophysical studies on purified components with the complexity of intact biological systems.

## Supplementary Information

Below is the link to the electronic supplementary material.

Supplementary file1

## Data Availability

Data are available on request to the corresponding author.
