# Supplementary material for: Measuring bacterial oxygen consumption rate to probe metabolic signature and antimicrobial susceptibility
Source: Eur Biophys J. 2026 Mar 28;55(3):487–98. doi: 10.1007/s00249-026-01834-7 (PMC13319873; doi:10.1007/s00249-026-01834-7)
Supplement: Supplementary file 1 — Supplementary file1 [file 249_2026_1834_MOESM1_ESM.docx]

MEASURING BACTERIAL OXYGEN CONSUMPTION RATE TO PROBE METABOLIC SIGNATURE AND ANTIMICROBIAL SUSCEPTIBILITY

Chiara Scribani Rossi^1§^, Simone Angeli^1§^, Bruno Casciaro^1^, Maria Rosa Loffredo^1^, Maria Luisa Mangoni^1^, Sharon Spizzichino^1^, Giovanna Boumis^1^, Manuel Espinosa-Urgel^2^, Marzia Arese^1^, Alessio Paone^1^§, Francesca Cutruzzolà^1^*, Serena Rinaldo^1^§*

^1^Department of Biochemical Sciences “A. Rossi Fanelli”, Sapienza University of Rome. Rome, Italy.

^2^Department of Biotechnology and Environmental Protection. Estación Experimental del Zaidin, CSIC. Granada, Spain.

§These authors contributed equally to the work

**Supplementary Materials.**


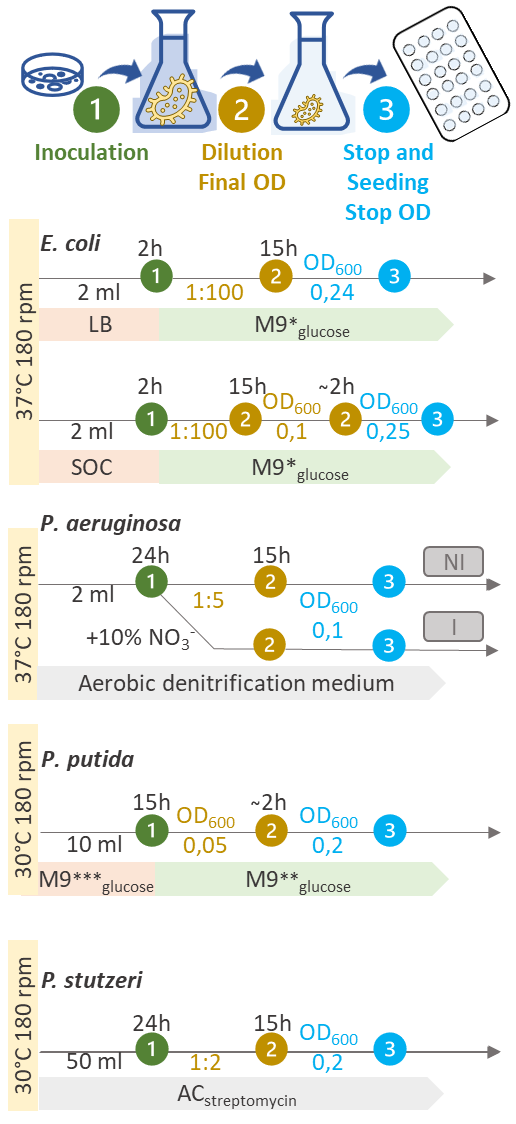


**Figure S1.** Growth timeline for each strain/condition assayed in this work. SOC inoculum for *E. coli* was used in the hypoxic experiments (and in the corresponding normoxic control, Figure 2D in the main manuscript). Growth phases are numbered in the Figure. OD reported in ocher refers to the final value obtained upon dilution (step 2); OD reported in the dilution step, in sky blue refers to the value of the growth immediately before seeding for Seahorse measurements. I and NI in the Figure refer to “Induced” and “Not induced” samples, respectively.

**
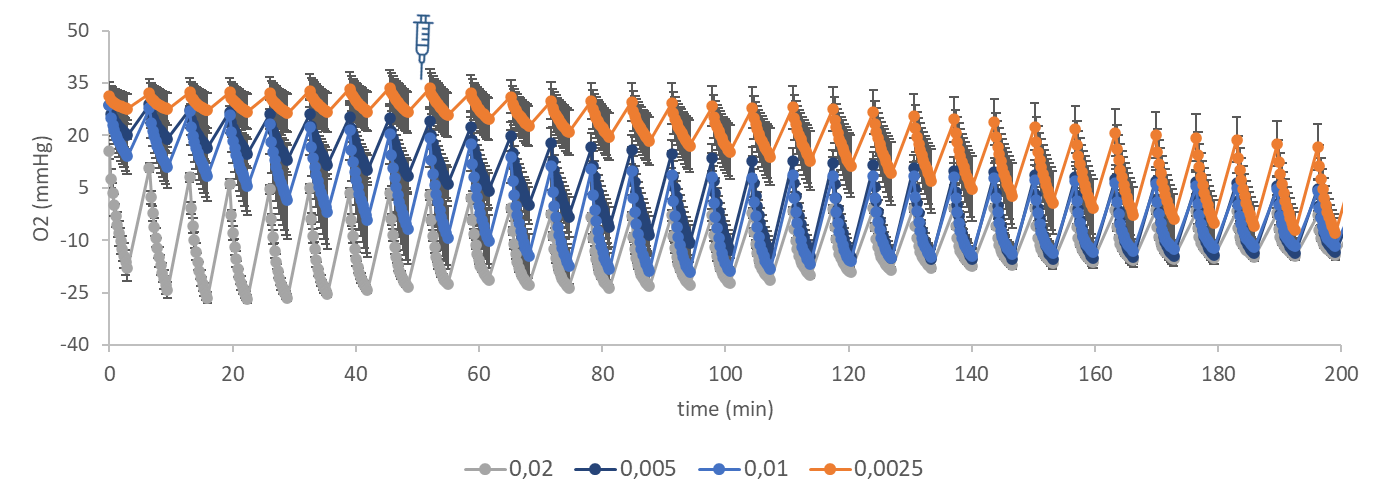
**

**Figure S2.** Respiratory activity under hypoxic conditions (5% O_2_). Oxygen consumption of different dilution of seeded *P. putida* (OD_600_ 0.02; 0.01; 0.005; 0.0025). Normoxic experiments were reported with 0.02 seeding ^1^; under hypoxic conditions, 0.0025 dilution is useful to follow respiratory activity without oxygen depletion in the microchamber (negative values).

Oxygen levels were measured every 3 min alternated with 3 min of mixing for gas exchange (see Figure S1 for technical details); the syringe indicates the point at which Carbon source (or control) was injected by the instrument (i.e. arginine).


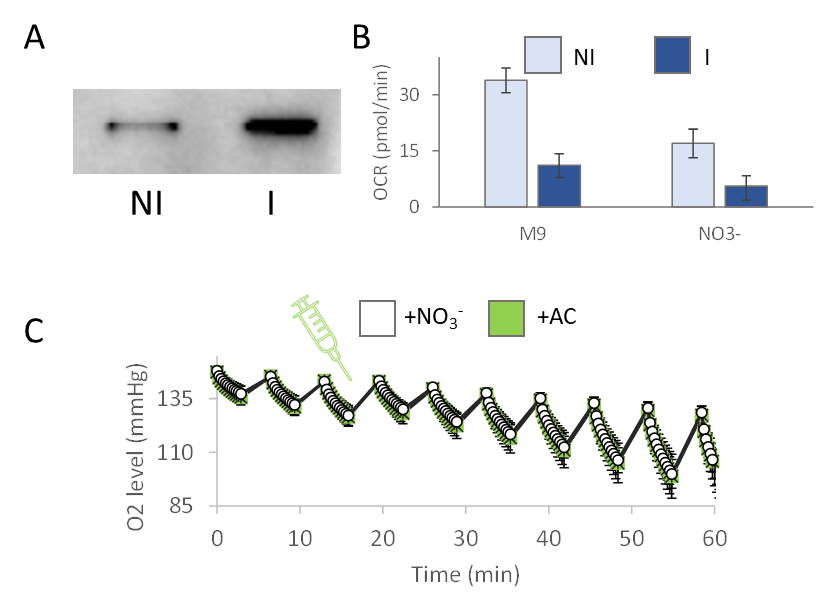


**Figure S3.** (A) Western blot with anti-cd_1_NiR antibody, targeting the respiratory nitrite reductase of *P. aeruginosa* PA14. The expression of the protein is present in the non-induced sample (“NI”) and is increased in the NO_3_^-^-treated sample (induced, “I”). (B) Comparison of the basal respiration (18’) of *P. aeruginosa* PA14 “NI” and “I” samples seeded with M9 (as reported in Figure 3A) or with NO_3_^-^. (C) Effect of NO_3_^-^ supplementation on the OCR of *P. stutzeri* MK21 strain; addition of NO_3_^-^ did not affect the OCR, as seen for *P. aeruginosa*.

**Figure S4.** GFP fluorescence in *P. putida* biofilms. Panel A displays images of the wild-type strain KT2442-GFP biofilm at three different initial optical densities: 0.09, 0.045, and 0.0225. Panel B presents images of the mutant strain ΔlapAΔlapF-GFP, in which the genes encoding for two key adhesins have been deleted. The images clearly demonstrate that the mutant strain exhibits a reduced capacity for biofilm formation compared to the wild-type

**Supplementary Methods.**

*Bacterial strains and growth:*

*P. putida* KT2440 was grown in M9* medium supplemented with 0.1 mM CaCl_2_ (M9**) plus 0.5 mM FeCl_2_ (M9***) and 5 mM glucose (the last two nutrients added as filtered solutions to the autoclaved medium). *P. stutzeri* ATCC 14405 MK21 (the spontaneous streptomycin-resistant mutant) is grown in asparagine-citrate medium (hereinafter AC), according to ^2,3^: NaCl, 20 g/L; MgSO_4_ · 7H_2_O, 2 g/L; Na-citrate · 2H_2_O, 7 g/L; KH_2_PO_4_, 2 g/L; L-Asparagine · H_2_O, 2 g/L; CaCl_2_ · 2H_2_O, 0.1 g/L; pH 6.8 plus 0.1 % Fe-Cu stock solution (composed by FeCl_3_, 20 g/L; CuCl_2_ · 2H_2_O, 0.17 g/L plus 1% HCl 1M; daily prepared and filtered). *P. putida* was inoculated into 10 ml of its growth media at 30°C and 180 rpm. The overnight culture was diluted to a final OD_600_=0.05 into 10 mL of M9*plus CaCl_2_ and glucose and grown at 30 °C, 180 rpm until an OD_600_ ~ 0.2 was reached. *P. stutzeri* was inoculated into 50mL of AC media plus 200ug/ml of streptomycin at 30°C and 180 rpm. After 24h the culture was diluted 1:2 in fresh media and let to grow overnight at 30°C and 180 rpm until OD_600_=0.2.

*Crystal violet staining:*

After cell attachment to the PLL-coated Seahorse plate by centrifugation, the supernatant was removed and 80 μL of 0.05% Crystal Violet was added to verify the amount of adhered cells. The Crystal Violet 0.05% stock solution was prepared by dissolving 50 mg of Crystal Violet (C6158 Sigma Aldrich) in 2.5 mL EtOH and 97.5 mL H_2_O. After 15 minutes at room temperature, the Crystal Violet was removed, the wells were rinsed twice with 90 μL of H_2_O and then 80 μL of 33% Acetic Acid is added. The solution became violet and after 15 minutes 70 μL was transferred to another plate. The intensity of the violet staining was quantified at 600 nm using the SpectraMax Mini plate reader (Molecular Device).

**Supplementary References.**

(1) Scribani-Rossi, C.; Molina-Henares, M. A.; Espinosa-Urgel, M.; Rinaldo, S. Exploring the Metabolic Response of Pseudomonas Putida to L-Arginine; **2024**. https://doi.org/10.1007/5584_2024_797.

(2) ZUMFT, W. G.; BRAUN, C.; CUYPERS, H. Nitric Oxide Reductase from Pseudomonas Stutzeri Primary Structure and Gene Organization of a Novel Bacterial Cytochrome Bc Complex. *Eur J Biochem* **1994**, *219* (1–2). https://doi.org/10.1111/j.1432-1033.1994.tb19962.x.

(3) Arese, M.; Zumft, W. G.; Cutruzzolà, F. Expression of a Fully Functional Cd1 Nitrite Reductase from Pseudomonas Aeruginosa in Pseudomonas Stutzeri. *Protein Expr Purif* **2003**, *27* (1). https://doi.org/10.1016/S1046-5928(02)00600-9.
